# Flame-Retardant Mechanism and Mechanical Properties of Wet-Spun Poly(acrylonitrile-*co*-vinylidene chloride) Fibers with Antimony Trioxide and Zinc Hydroxystannate

**DOI:** 10.3390/polym12112442

**Published:** 2020-10-22

**Authors:** Ji Su Kim, Ji Eun Song, Daeyoung Lim, Heejoon Ahn, Wonyoung Jeong

**Affiliations:** 1 Human Convergence Technology R&D Department, KITECH, Ansan 15588, Korea; nrhope2@kitech.re.kr (J.S.K.); mplala@kitech.re.kr (J.E.S.); zoro1967@kitech.re.kr (D.L.); 2 Department of Organic and Nano Engineering, Hanyang University, Seoul 04763, Korea; ahn@hanyang.ac.kr

**Keywords:** flame retardant mechanism, poly(acrylonitrile-co-vinylidene chloride), anitimony trioxide, zinc hydroxystannate, flame retardancy

## Abstract

To produce flame retardant poly(acrylonitrile-co-vinylidene chloride) (PANVDC) fibers with limiting oxygen index (LOI) values above 28%, flame retardants are added to fibers. Because antimony trioxide (ATO) used widely for PANVDC is suspected as a carcinogen, non-toxic zinc hydroxystannate (ZHS) could be the alternative for reduction of ATO usage. Moreover, a flame retardant efficiency of the combination of ATO with ZHS could be expected because it was reported that ATO resists flame in the gas phase, whereas ZHS reacts in the condensed phase. Therefore, this study discussed the flame retardant mechanisms of ATO and ZHS in PANVDC, and evaluated the efficiency of the combination. PANVDC fibers with ATO and ZHS in 15 phr were produced by wet spinning. When ZHS was added, a more cyclized structure was detected (e.g., 1-methylnaphthalene) through pyrolysis−gas chromatography-mass spectrometry (Py-GC/MS). As a result of SEM-EDX analysis, Sb and Cl hardly remained in char layers of PANVDC-ATO; meanwhile, Zn, Sn, and Cl remained in that of PANVDC-ZHS. This implied that SbCl_3_ from reaction of ATO and HCl reacts in the gas phase, whereas ZnCl_2_ and SnCl_2_ from ZHS and HCl promotes the cyclization reaction of PANVDC in the condensed phase. The LOI values of PANVDC, PANVDC-ATO, and PANVDC-ZHS were 26.4%, 29.0%, and 33.5%, respectively. This suggests that ZHS is a highly effective for PANVDC. Meanwhile, the LOI of PANVDC containing ATO-ZHS mixture is 31.0%. The combination of ATO and ZHS exhibited no efficiency. The addition of ATO and ZHS slightly reduced the tenacities of the fibers, respectively, 3.11 and 3.75 from 4.42 g/den.

## 1. Introduction

Modacrylic fibers are defined as those fibers containing 35–85 wt.% acrylonitrile (AN) units. Poly(acrylonitrile-co-vinylidene chloride) is one of the copolymers synthesized for modacrylic fibers [1]. Although modacrylic fiber has flame retardancy with a limiting oxygen index (LOI) value above 26% compared to ordinary acrylic fiber with LOI 18–20%, flame retardants are usually added to produce commercial flame retardant modacrylic fiber [2]. The flame retardant-added modacrylic fibers can be used as flame-resist textile which is generally defined with LOI value above 28%. This modacrylic fiber has excellent touch and cost advantage so that it can be useful to flame resistant clothing.

Antimony trioxide (ATO, Sb_2_O_3_) is one of the most common flame retardants added to modacrylic fiber [2]. ATO is a representative flame retardant synergist that shows excellent flame retardant performance when used with a halogen-based polymer such as poly(vinyl chloride) (PVC). Although ATO is an efficient flame retardant, antimony and its compounds are suspected of being carcinogenic to humans. Therefore, alternative additives have been studied.

Various researches have been conducted so far to reduce usage of antimony oxide for halogen compounds. Some hydroxides, such as magnesium hydroxide or alumina trihydrate, are used as flame retardants for PVC due to the benefit of their low cost [3]. Nanofillers such as hydrotalcite or some hydroxides act as an efficient flame retardant at low addition levels in PVC [4]. Phosphorus-containing flame retardants are also used as flame retardant plasticizer for PVC [5]. Zinc compounds (zinc borate, zinc stannate, and zinc hydroxystannate) have good synergistic effect with halogen compounds through enhancement of char formation ZHS in a condensed phase unlike ATO [6,7].

Zinc hydroxystannate (ZHS, ZnSn(OH)_6_) is one of the alternative flame retardant to ATO. Qu et al. reported results that a LOI value for ZHS was 31.6% at 15 phr levels in a plasticized PVC formulation. In addition, Xu et al. reported that the LOI value for ZHS was higher than that for ATO at 15 phr levels in a flexible PVC [8]. Moreover, the tin (Sn) in ZHS exists as a biologically inert inorganic substance that is harmless to humans [9]. Although there has been a lot of research about ZHS for PVC, few studies have used ZHS to improve the flame retardancy of PANVDC. Tanaka et al. used ZHS for PANVDC fiber and reported that the LOI value was 29.2%. However, this result was that of blended fabric consisting 60 wt.% of PANVDC and 40 wt.% of cotton fiber [10]. Song et al. also applied ZHS to PANVDC, but they focused on improving compatibility of ZHS in PANVDC [11]. Thus, the flame retardant mechanism of PANVDC with ZHS has rarely been studied. The flame retardant mechanism of ZHS in PVC was reported by Xu et al. through quantitative analysis of the residual char [7]. They suggested that ZnCl_2_ from the reaction of ZHS and HCl acted as a catalyst of dehydrochlorination and promoted early crosslinking, leading to rapid charring. ZHS would be expected to act same in PANVDC which also contains chlorine. Because the flame retardant mechanism of ZHS differs from that of ATO, a combination of ZHS and ATO would be expected to be efficient on flame retardancy. Qu et al. reported the LOI value for combination of ATO and ZHS was 40.4% at 15 phr levels. This value was much higher than that of a neat plasticized PVC: 25.2% [8].

Therefore, the purpose of this study is to discuss the flame retardant mechanism of PANVDC fibers with the different type of flame retardant, such as (1) ATO and (2) ZHS, respectively. It has been rarely reported that the flame retardant efficiency of the combination of ATO with ZHS for PANVDC fiber. For this, the different flame mechanism of ATO and ZHS in PANVDC was analyzed by pyrolysis−gas chromatography-mass spectrometry (py-GC/MS), energy dispersive X-ray spectroscopy (SEM-EDX), and thermogravimetric analysis (TGA). Moreover, the efficiency of the combination of ATO with ZHS for PANVDC fiber was examined by the flame retardancy analysis such as LOI and microscale combustion calorimetry (MCC), respectively. There were barely observed the flame retardant efficiency of the combination of ATO and ZHS. The influences of ATO and ZHS on the mechanical properties of the PANVDC fibers were also investigated. The addition of flame retardants decreased the tenacities and elongations of the fibers slightly.

## 2. Experimental

### 2.1. Materials

Antimony trioxide (99.83%, ATO, Sb_2_O_3_) was purchased from Sooyangchemtec Co., Ltd. (Yesan, Korea). Zinc hydroxystannate (99.8%, ZHS, ZnSn(OH)_6_) was obtained from Ditto Technology Co., Ltd. (Gunpo, Korea). The PANVDC copolymer was a commercial flame retardant modacrylic with an AN to VDC compositional molar ratio of 67:33. The number average molecular weight and polydispersity of PANVDC were 214.000 g/mol and 2.56, respectively. Dimethyl sulfoxide (99.9%, DMSO) was obtained from Samchun Chemical Co., Ltd. (Seoul, Korea).

### 2.2. Methods

#### 2.2.1. Wet Spinning of PANVDC Fibers with Flame Retardants

**Figure 1:**
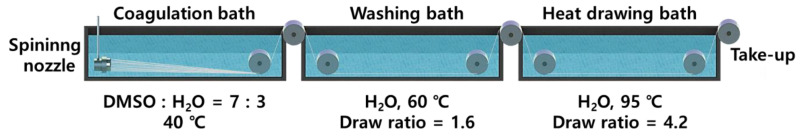
Wet spinning system used in this study.

**Figure 2:**
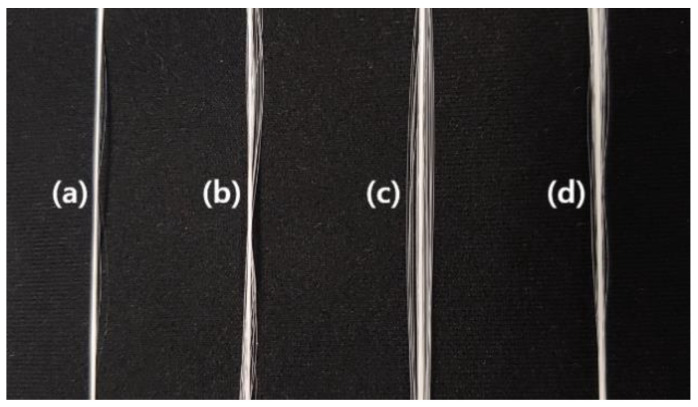
The fibers obtained through wet spinning: (**a**) PANVDC, (**b**) PANVDC-ATO, (**c**) PANVDC-ATO/ZHS, and (**d**) PANVDC-ZHS fiber.

The fibers were prepared via a lab-scale wet-spinning system. The detailed process is as follows. Figure 1 presents a schematic diagram of the spinning equipment. DMSO was used as a solvent because it is relatively less toxic than other solvents such as N,N-dimethylmethanamide and acetone. PANVDC was dissolved in DSMO to a concentration of 34 wt.%, in which ATO and ZHS were dispersed using an ultrasonic bath (Power sonic 420, Whashin Tech Co., Gyeonggi, South Korea) for 20 min. The spinning solutions were deaerated, filtered (400 mesh), and spun into a DMSO/H_2_O coagulation bath (70/30 *w/w*) using a 100 filament spinneret with 70 μm capillary diameters. The temperature of the spinning solution was 50 °C, and the spinning speed was 1.22 mL/min. First, the solution was spun into a coagulation bath at 40 °C. After the coagulation bath, the fibers were passed through a washing bath at 60 °C, followed by a hot stretching bath at 95 °C. The fibers were finally dried in a 60 °C convection oven for 24 h. The fibers were finally produced as shown in Figure 2.

#### 2.2.2. Preparation of PANVDC Films with Flame Retardants

To evaluate the flame retardancy through the limiting oxygen index (LOI) test, the PANVDC films containing the flame retardants were prepared using a solution casting method. The detailed process is as follows. PANVDC (8.7 g) was dissolved in DMSO (21.3 mL), in which ATO and ZHS were dispersed previously by ultra-sonication. The solution was cast onto a glass substrate, pilled, and dried at 100 °C for 1 h to form a casting film. The PANVDC films produced included 15 phr of flame retardants.

#### 2.2.3. Characterization

- Flame Retardant Mechanisms

Py-GC/MS was used to measure the pyrolysis products and molecular structures of fibers. Py-GC/MS was conducted on a pyroprobe (PY-2020iD, Frontier, Fukushima, Japan) combined with a gas chromatograph (7890, Agilent, Santa Clara, CA, USA) and mass spectrometer (5975, Agilent, Santa Clara, CA, USA). The pyrolysis temperature ranged from room temperature to 600 °C in a nitrogen atmosphere at a heating rate of 20 °C/min. After pyrolysis, the volatile products were sent to the GC injector with a set temperature of 320 °C. The char surface of samples was examined using scanning electron microscopy joined with energy dispersive X-ray spectroscopy (SEM-EDX) (SU8000, Hitachi, Tokyo, Japan). Elemental maps were obtained for Cl, Sb, Zn, and Sn based on the EDX analysis.

- Thermal Properties

Thermogravimetric analysis (TGA, TA instrument Q500, DE, USA) was performed under air flow at a heating rate of 20 °C/min to 800 °C.

- Flame Retardancy

The vertical burning tests were conducted according to the UL 94 (Underwriter’s Laboratory, thin material vertical burning test) under controlled laboratory conditions. The UL 94 test is performed with a 20 mm vertical flame by twice contacting a molded sample with dimensions of 125 × 13 × 0.06 thickness in mm for 10 s. To pass UL 94 V2, specimens must not burn with flaming or glowing combustion up to the specimen holding clamp. For UL 94 V0, the flame should extinguish within 10 s after each ignition, with less than 50 s as total burn for 5 samples and no burning drips. The limiting oxygen index (LOI) was measured on films (140 mm × 52 mm × 0.5 mm) using the KS M ISO 4589-2, 2016 method. Microscale combustion calorimetry (MCC) test was conducted using a Pyrolysis Combustion Flow Calorimeter (Fire Testing Technology Ltd., East Grinstead, UK), according to ASTM D 7309—method A. Fiber samples (5 mg) were exposed at a heating rate of 1 °C/s to 850 °C in a stream of nitrogen flowing at 80 cc/min and then an O_2_ flow rate of 20 cc/min was mixed.

- Morphology and Mechanical Properties of Fibers

The fiber cross-sectional morphology was examined by backscattered electron scanning electron microscopy (BSE-SEM, JEOL Ltd., JSM6700F, Tokyo, Japan). For the cross section morphology, the fibers were immersed in liquid nitrogen and fractured carefully. The tensile properties of single fibers were tested using a Favimat fiber test system (Textechno H., Favimat-airobot2, Mönchengladbach, Germany). At least twenty fibers were subjected to a tensile test at a cross-head speed of 20 mm/min and a gauge length of 20 mm. The orientation of polymer chain crystal in fbers was determined using 2D wide-angle X-ray diffraction (WAXD, Bruker D8 Discovery, Karlsruhe, Germany). WAXD was performed using Cu Kα radiation (λ = 0.1542 nm) at a voltage and current of 50 kV and 100 μA, respectively. The 360 °C azimuthal circle was used to permit the fiber axis to rotate 360 °C about the vertical. The orientation was calculated from the equation

(1) $$\mathrm{Crystal}\;\mathrm{orientation}\;=\;\frac{360-\mathrm{FWMH}}{360}\;\times 100\;\left(\%\right)\;\;\;$$

where FWMH is the half-value width in degrees of the curve of intensity against azimuthal angle [12].

## 3. Results and Discussions

### 3.1. Preparation of PANVDC Fiber with Flame Retardants

Table 1 shows the mechanical properties of fiber and UL 94 results according to the ZHS content. With the addition of ZHS, both tenacity and elongation decreased compared to that of neat PANVDC fiber. The tenacity of the 10, 15 and 20 phr ZHS added fiber was 3.98, 3.75, and 3.46 g/den, respectively. As the ZHS content increased, tenacity and elongation decreased. The vertical burning test is used widely to evaluate the relative flammability of polymeric materials. Neat PANVDC samples burned immediately when exposed to the flame, and all specimens were entirely burned down. For 10 phr ZHS addition, specimens ceased combustion when the flame was removed. However, they were already burned up to the holding clamp. Therefore, neat PANVDC and 10 phr ZHS added PANVDC were not able to be graded. In the case of 15 and 20 phr addition, they both ceased burning immediately once the flame was removed. Moreover, they were not burned up to the holding clamp and graded V-0. In the present study, the content of flame retardants was determined to be 15 phr, considering mechanical properties and flame retardancy of the PANVDC fibers. The experimental samples listed in Table 2. The PANVDC fibers containing ATO and ZHS are referred to as PANVDC-ATO and PANVDC-ZHS, respectively. The PANVDC fibers containing a mixture of ATO and ZHS at a 50:50 ratio is referred to as PANVDC-ATO/ZHS.

### 3.2. Flame Retardant Mechanisms

Table 3 lists the Py-GC/MS result of PANVDC fibers containing ATO and ZHS. In the decomposition products of all fibers, HCl and chloropyridine isomers were detected as the major products. HCl plays an important role in the flame-retardant mechanism of halogen compounds [13]. Pyridine is thought to be resulted from the formation of a stable carbonized structure in polyacrylonitrile (PAN) [14]. The decomposition products of PANVDC and PANVDC-ATO fibers were the same except for the antimony compounds. When ZHS was added to the fibers, the decomposition products changed, even though the ZHS content ratio was relatively small, such as PANVDC-ATO/ZHS fibers. 2-Pentenedinitrile, 3-methylbenzonitrile, and 3-chlorobenzonitrile were detected as the pyrolysis products of the PANVDC and PANVDC-ATO fibers, but they were not observed in the fibers containing ZHS. Acetonitrile, benzonitrile, m-toluidine, and 1-methylnaphthalene were detected as a decomposition product of only fibers with ZHS, PANVDC-ATO/ZHS, and PANVDC-ZHS. ZHS appeared to impart flame retardancy through a different reaction from that of pure PANVDC. Acetonitrile produced by the decomposition of PANVDC fibers containing ZHS was attributed mainly to the cyclization of PAN [15]. As a result, aromatic compounds, such as benzonitrile, instead of 2-pentenedinitrile which is a highly flammable liquid, were observed. Moreover, m-toludine was detected in the pyrolysis materials of the fibers including ZHS. The breaking of carbon–nitrogen bonds and the rearrangement of the polycyclic structure will lead to the formation of aromatic compounds, which would react further with the remained nitrile groups [15]. Therefore, 1-methylnaphthalene was observed instead of 3-methylbenzonitrile and 3-chlorobenzonitrile. During decomposition–carbonization, the cyclic structure began to carbonize, forming a stable polycyclic structure with dehydrogenation. Overall, these aromatic substances produced in the pyrolysis process appeared to work significantly in forming a dense char layer and impart excellent flame retardancy to PANVDC [15,16,17].

**Figure 3:**
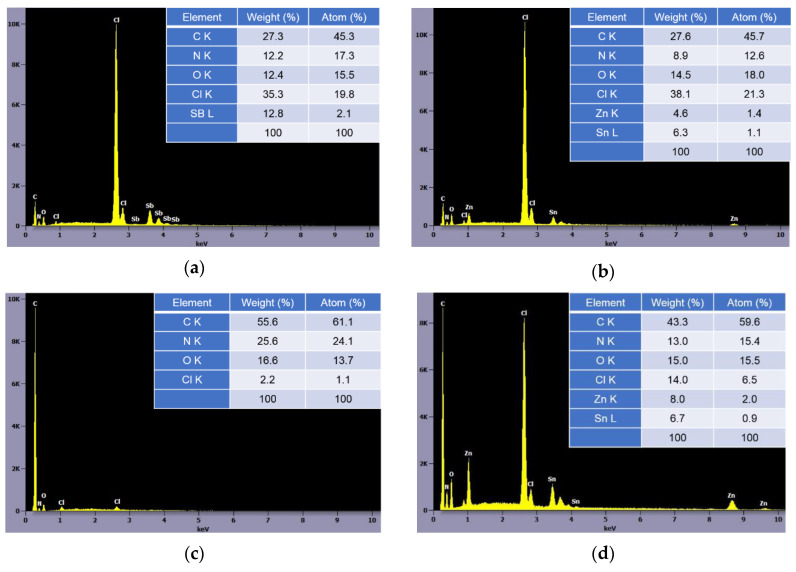
The EDX results of the surface of (**a**) PANVDC-ATO and (**b**) PANVDC-ZHS films and the char surface of (**c**) PANVDC-ATO and (**d**) PANVDC-ZHS films.

**Figure 4:**
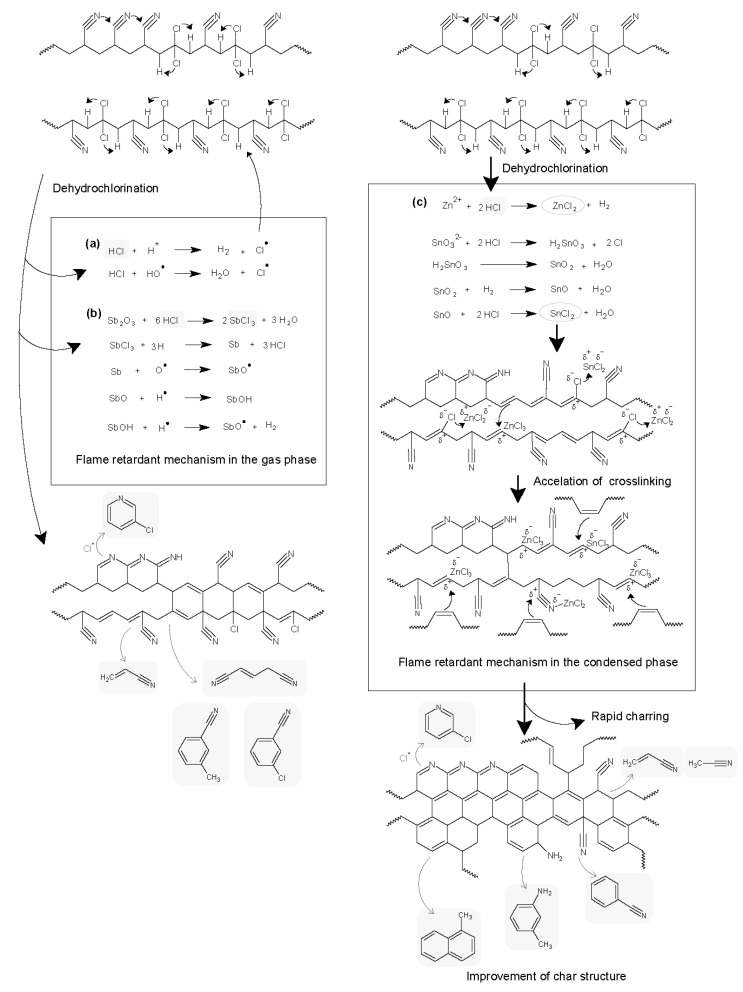
The flame retardant mechanism of (**a**) PANVDC, (**b**) PANVDC-ATO, and (**c**) PANVDC-ZHS.

Figure 3d suggested that ZnCl_2_ and SnCl_2_ from reaction of ZHS and HCl remained to promote cross-liking in the solid phase [7]. ZnCl_2_ and SnCl2 are believed to promote the cyclization reaction of the acrylonitrile units. On the other hand, Figure 3c implied that ATO with HCl acts as flame retardant mainly in the gas phase. ATO rarely participated in promoting the reaction of AN-units directly. The different flame retardant mechanisms of PANVDC with ATO and ZHS were illustrated in Figure 4. Few differences were observed between the decomposition products of the ZHS only added fibers and the ATO-ZHS mixture added fibers, suggesting that there were no special interactions between ATO and ZHS. The different flame retardant mechanisms of ATO and ZHS in PANVDC are illustrated in the Figure 4.

### 3.3. Thermal Properties

**Figure 5:**
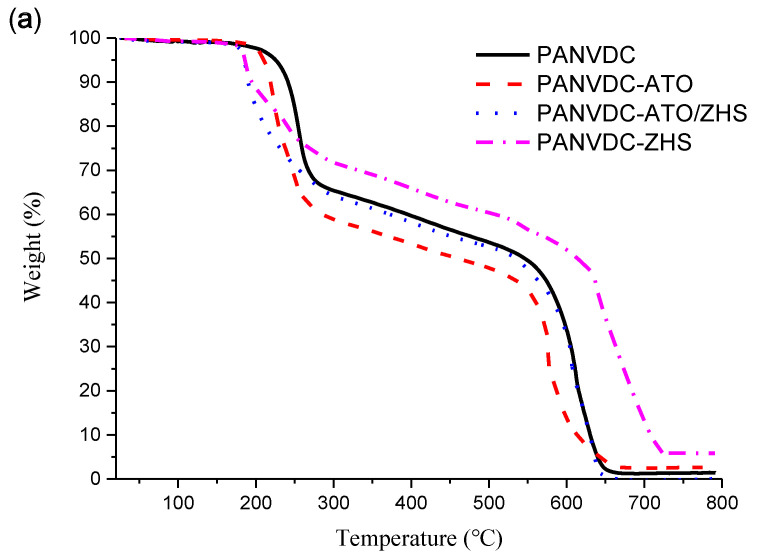

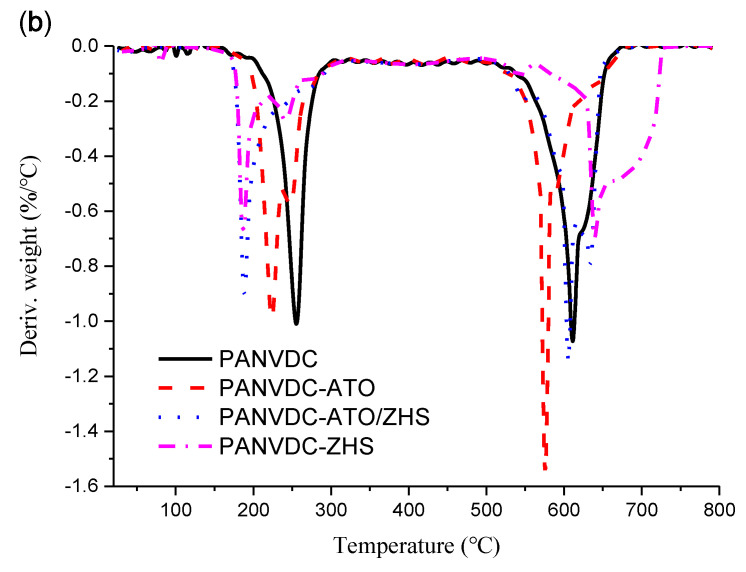
(**a**) Thermogravimetric analysis (TGA) and (**b**) derivative thermogravimetric (DTG) curves of the PANVDC fibers with flame retardants at a heating rate 20 °C/min to 800 °C under air conditions.

Figure 5 and Table 4 present the TGA and derivative thermogravimetric (DTG) curves and results of the PANVDC fibers with flame retardants. The analysis was performed under air conditions at a heating rate of 20 °C/min to 800 °C. The pyrolysis process of the fibers under air conditions was analyzed by dividing it into two stages according to the rapid change in the rate of mass loss (%/°C) [18,19].

The first stage appeared to result from dehydrochlorination and the subsequent reactions [13]. The TMR1 of the fibers, including flame retardants, was lower than that of the neat PANVDC fiber. This indicates that the flame retardants promoted the dehydrochlorination reaction. The TMR1 of the fibers containing ZHS was much lower than that of PANVDC-ATO, which was almost 70 °C lower than that of PANVDC. ZHS is a powerful accelerator for the dehydrochlorination of PANVDC. In the first stage, the mass loss of PANVDC-ATO was the largest among those of fibers, whereas that of PANVDC-ZHS was the smallest. This was attributed to the different properties of materials that play an important role in the flame retardant mechanism of ATO and ZHS. ATO reacts with HCl to produce SbCl_3_. The boiling point of SbCl_3_ is 220 °C, i.e., it is volatile. SbCl_3_ is an effective free radical interceptor in the gas phase of the combustion process [20]. On the other hand, ZnCl_2_ is the reaction product of ZHS and HCl, which is barely volatile with a boiling point of 732 °C. ZnCl_2_ is a strong Lewis acid that promotes the crosslinking of polymers in the solid phase [7]. Therefore, it appears that the mass loss of PANVDC-ATO is greater than that of PANVDC, whereas that of PANVDC-ZHS is smaller.

The second stage appears to be caused by the decomposition of the char layer produced by the subsequent reaction after dehydrochlorination [10]. The TMR2 of PANVDC-ZHS was 640 °C, the highest among the fibers. On the other hand, the TMR2 of PANVDC-ATO was 576 °C, the lowest among the fibers. This result was quite different from the first stage. This suggests that the char layer formed in PANVDC-ZHS had a stable carbonized structure that was more difficult to decompose by heat and oxidation than that of the char layer in PANVDC-ATO. Liu et al. reported that for polymers with a good char formation, more fragments could be kept in the condensed phase and fewer are released into the gas phase during combustion [21]. Therefore, it implied that the dense char structure of PANVDC-ZHS could suppress smoke-release.

The TMR1 of PANVDC-ATO/ZHS was 188 °C, indicating that the dehydrochlorination was promoted early, as in PANVDC-ZHS. On the other hand, the mass loss (%) at the first stage and the TMR2 were similar to those of PANVDC. Therefore, the char layer of PANVDC-ATO/ZHS was thought to have a similar level of stability to that of the char layer of PANVDC.

### 3.4. Flame Retardancy of PANVDC Containing ATO and ZHS

LOI is an important measure for evaluating the flame retardancy of polymeric materials. The LOI test was performed using a film-type PANVDC; Table 5 lists the results. Although the pyrolysis products of PANVDC and PANVDC-ATO were similar, the LOI value of PANVDC-ATO was higher than that of PANVDC. ATO promotes only the original flame retardant reaction of a pure PANVDC copolymer. When a mixture of ZHS and ATO was added, there was no dramatic increase and decrease in the LOI value compared to that of PANVDC-ATO and PANDVC-ZHS. This suggests that the combination of ATO and ZHS had not any efficiency on the flame retardancy of PANVDC fibers. The LOI value of PANVDC-ATO/ZHS was higher than that of PANVDC-ATO. The LOI value of PANVDC-ZHS was the highest among the experimental samples. Therefore, ZHS is a more efficient flame retardant with a PANVDC than ATO.

**Figure 6:**
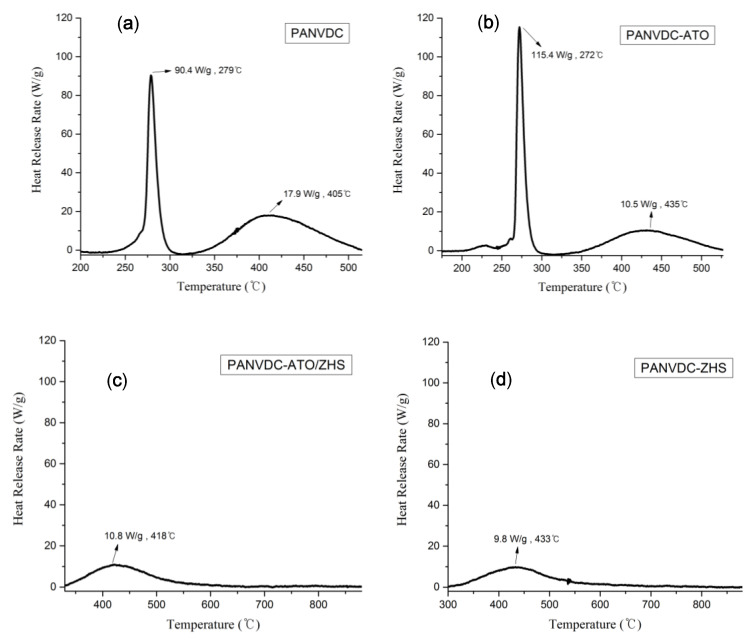
The heat release rate versus temperature curves of PANVDC fibers: (**a**) PANVDC, (**b**) PANVDC-ATO, (**c**) PANVDC-ATO/ZHS, and (**d**) PANVDC-ZHS.

The flame retardancy of the PANVDC fibers was evaluated through MCC. Table 6 lists the relative parameters obtained from the MCC test, such as the peak heat release rate (pHRR), temperature at the peak heat release rate (TPHRR), total heat release rate (THR), and heat release capacity (HRC). Figure 6 shows the heat release rate versus temperature curves of the fibers. The PHRR is a parameter determining the behavior of fires [15,22]. The PHRR of PANVDC fibers containing ZHS was observed at higher temperatures. The PHRR of PANVDC-ATO/ZHS and PANVDC-ZHS fibers were significantly lower than that of PANVDC and PANVDC-ATO fibers. This suggests that ZHS had great flame-resist performance in the fiber. Therefore, ZHS can be used as more efficient flame retardant for PANVDC than ATO.

### 3.5. Morphology and Mechanical Properties of Fibers

**Figure 7:**
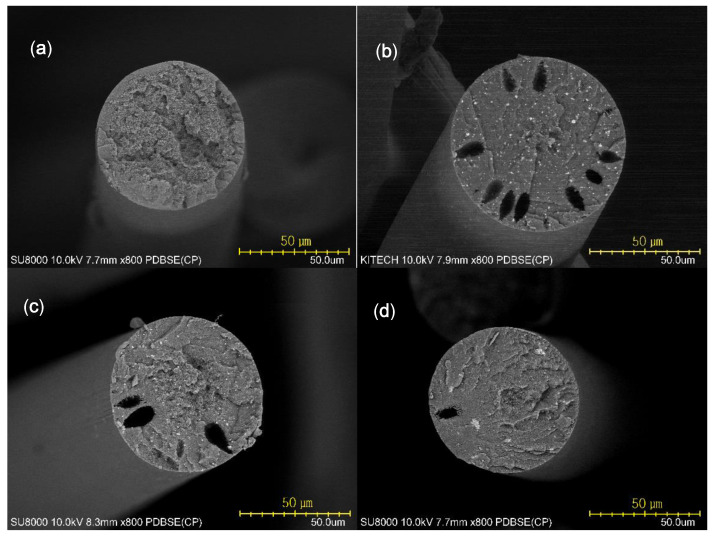
BSE-SEM images of the coagulated fiber cross section: (**a**) PANVDC, (**b**) PANVDC-ATO, (**c**) PANVDC-ATO/ZHS, and (**d**) PANVDC-ZHS.

**Figure 8:**
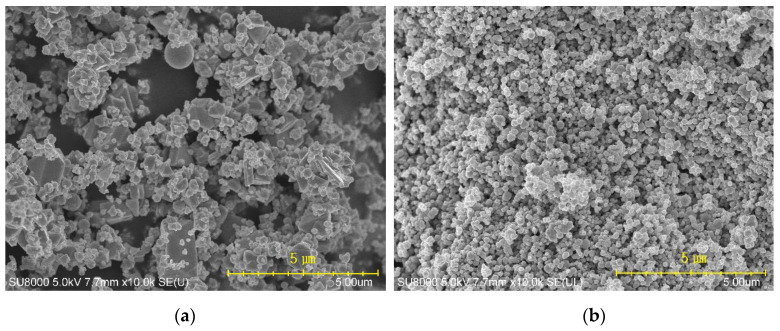
SEM images of the powder particles of (**a**) ATO and (**b**) ZHS.

Figure 7 shows SEM images of the coagulated fibers cross sections using backscattered electrons (BSE). The PANVDC fibers with the flame retardants had a round cross section with some pores, while the cross section of PANVDC fibers had no pores. There were several pores in the fibers containing ATO. More pores were observed in the PANVDC-ATO fibers than the PANVDC-ATO/ZHS. This suggests that ATO, which had a large particle size (~2 μm) as shown in Figure 8a, interfered with the diffusion of the solvent in and out of the fibers during coagulation, resulting in pores in fibers. The white dots in the SEM images were attributed to ATO and ZHS particles, which were distributed in the cross section of the PANVDC fibers with flame retardants. This suggests that ATO and ZHS were barely eluted in coagulation bath, that is, DMSO/water solution at 50 °C and well introduced in the fibers. The agglomeration of particles in the PANVDC-ZHS fibers was observed. This appears to be the result of the aggregation of nano-sized ZHS particles (~250 nm) as shown in Figure 8b. 

**Figure 9:**
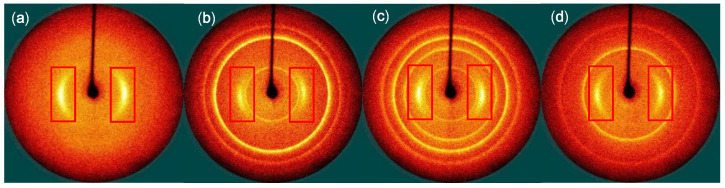
2D WAXD patterns of the drawn fibers: (**a**) PANVDC, (**b**) PANVDC-ATO, (**c**) PANVDC-ATO/ZHS, and (**d**) PANVDC-ZHS.

**Figure 10:**
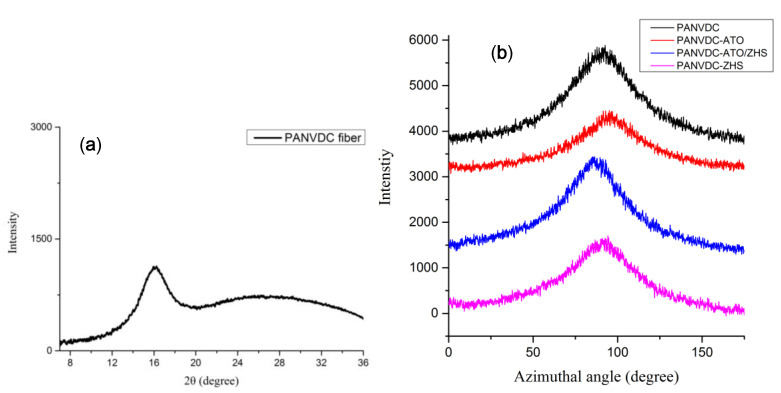
(**a**) The XRD graph of PANVDC fiber. (**b**) The azimuthal graph of PANVDC fiber with flame retardants.

Polymers can be oriented in specific direction because they have a long chain structure. Fibers have good physical properties when polymer chain crystals are oriented in the axial direction of the fiber. Therefore, the coagulated fibers were washed and drawn in hot water baths with a continuous process to remove the excess solvent and increase the molecular orientation of the PANVDC chains. Figure 9 presents 2D WAXD patterns of the drawn fibers. When the polymer chain crystals are oriented in fiber axial direction, they appear in a symmetrical arc shape in WAXD pattern. For the crystals without any orientation, they appear in a circle shape in the pattern [23]. The circular pattern in the flame retardant-added fiber seemed to be due to flame retardants. On the other hand, all fibers had symmetrically arc-shaped patterns indicating presence of polymer crystals. However, the differences of the arc-shaped patterns of the fibers were not observed clearly. In order to analyze the degree of orientation more clearly, it was calculated through azimuthal scan [12]. Because the peak of the PANVDC crystal appears at about 16° of 2theta in Figure 10a, we located the fiber at 16° of 2theta and the fiber axis was rotated 360° about the vertical using the 360° azimuthal circle. Then, the crystal orientation (%) of the fiber was calculated from equation (1) and listed in Table 7. The degree of orientation was slightly different for each fiber. Although the orientation of the PANVDC-ATO/ZHS fiber was 72.61%, higher than other fibers, this was not remarkable increase.

**Figure 11:**
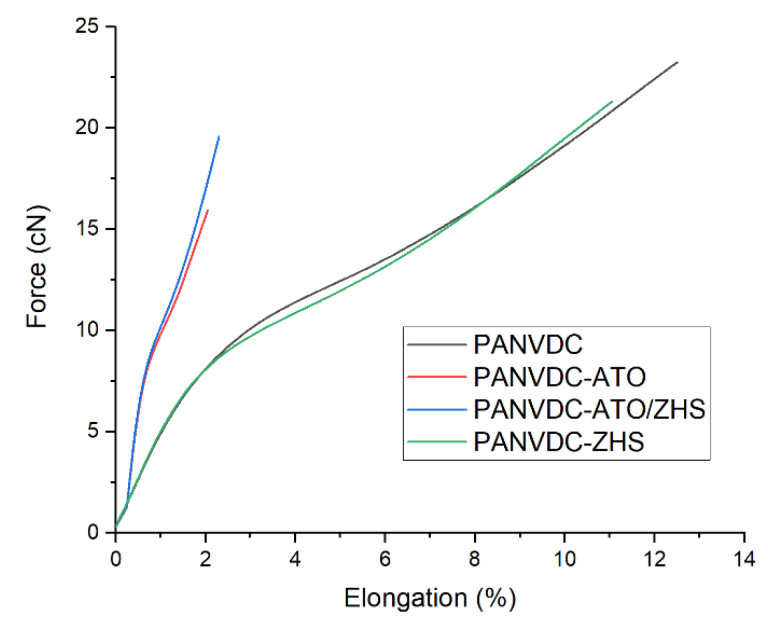
Average force/elongation-diagram of the drawn PANVDC fibers with the flame retardants.

Table 8 lists the mechanical properties of the drawn PANVDC fiber and PANVDC fibers with the flame retardants. The tenacity is tensile strength in relation to the linear density of the fibers. The tenacity and elongation of the PANVDC fibers containing flame retardants were lower than those of the PANVDC fibers. The particles of the flame retardants could lead to a defect that resulted from pores in fibers as shown in Figure 8. In Figure 11, the tensile strengths of PANVDC-ZHS and PANVDC fibers were relatively similar, but the tenacity of PANVDC-ZHS was lower than that of PANVDC fiber due to the higher linear density of PANVDC-ZHS fiber. Although the orientation of the PANVDC-ATO fiber was 71.08%, slightly higher than that of PANVDC-ZHS fiber (70.30%) as shown in Table 7, the tenacity of PANVDC-ATO was 3.11 g/den, lower than that of PANVDC-ZHS fiber (3.75 g/den). This suggests that many pores in the PANVDC-ATO fibers as shown in Figure 8b affected the mechanical properties of the fibers.

## 4. Conclusions

This study examined the flame retardant mechanism of PANVDC fibers according to the type of flame retardant, such as (1) ATO and (2) ZHS. py-GC/MS showed that the pyrolysis materials of the PANVDC and PANVDC-ATO fibers were the same except for the antimony compound. On the other hand, the pyrolysis material was similar to that of PAN with the addition of ZHS to PANVDC.

TGA revealed that the char layer of PANVDC fiber containing ZHS had a more stable carbonized structure against heat and oxidation than those of the char layer of PANVDC fiber and the fiber containing ATO. Therefore, the flame retardant mechanism of the PANVDC fibers containing ATO and ZHS is as follows.

- Flame retardant mechanism of the PANVDC-ATO fiber: ATO played a role in the original flame retardant mechanism of PANVDC, which resulted mainly from a halogen unit in the gas phase.
- Flame retardant mechanism of the PANVDC-ZHS fiber: ZHS reacted with the halogen unit and then with the AN unit to promote cyclization and carbonization, contributing to a stable char structure and flame retardancy.

The flame retardancy of PANVDC using a flame retardant was evaluated, and the results were as follows. The LOI value of PANVDC-ZHS was highest among the PANVDC films. As a result of MCC, the PHRR of PANVDC-ATO/ZHS and PANVDC-ZHS fibers were significantly lower than that of the PANVDC and PANVDC-ATO fibers. A stable char layer formed rapidly in PANVDC with ZHS, which contributed to flame retardancy effectively. Therefore, ZHS is a more efficient flame retardant for PANVDC than ATO.

Another purpose of the study was to investigate the efficiency of combination of ATO and ZHS for flame retardancy of PANVDC. The LOI value of PANVDC-ATO/ZHS was higher than that of PANVDC-ATO but lower than that of PANVDC-ZHS. The PHRR of PANVDC-ATO/ZHS was lower than that of PANVDC-ATO but similar with that of PANVDC-ZHS. Overall, no efficiency was observed in the combination of ATO and ZHS.

## Figures and Tables

**Table 1:** Mechanical properties and results of the vertical burning test (UL94) of PANVDC according to ZHS content.

ZHS Content (phr)	Tenacity (g/den)	Linear Density (den)	Elongation (%)	UL 94 Grade
0	4.42	5.39	12.52	-
10	3.98	5.33	11.17	-
15	3.75	5.81	11.05	V-0
20	3.46	5.64	10.63	V-0

Specimens burned with flaming combustion up to the specimen holding clamp.

**Table 2:** Experimental samples with flame retardants.

Sample Code	Polymer	Flame Retardants
PANVDC	PANVDC	None
PANVDC-ATO	PANVDC	ATO (15 phr)
PANVDC-ATO/ZHS	PANVDC	ATO/ZHS(50:50) (15 phr)
PANVDC-ZHS	PANVDC	ZHS (15 phr)

**Table 3:** Results of pyrolysis−gas chromatography-mass spectrometry (Py-GC/MS) of the PANVDC fibers with the flame retardants.

**PANVDC**		**PANVDC-ATO**	
**Retention Time (min)**	**Compound Identified**	**Retention Time (min)**	**Compound Identified**
1.469	hydrogen chloride *	1.469	hydrogen chloride *
1.679	acrylonitrile *	1.679	acrylonitrile *
1.908	methylacrylonitrile *	1.908	methylacrylonitrile *
2.923	2,4-pentadienenitrile	2.923	2,4-pentadienenitrile
4.313	cyanopentadiene	4.313	cyanopentadiene
5.319~5.466	chloropyridine isomers *	5.319~5.466	chloropyridine isomers *
5.694	2-pentenedinitrile	5.694	2-pentenedinitrile
7.404	2-methylenepentanedinitrile *	7.404	2-methylenepentanedinitrile *
7.651	2-methylpentanedinitrile	7.651	2-methylpentanedinitrile
7.917	3-methylbenzonitrile	7.917	3-methylbenzonitrile
8.529	3-chlorobenzonitrile	8.529	3-chlorobenzonitrile
-	-	9.856	Antimony compound
10.551	isophthalonitrile	10.551	isophthalonitrile
13.935	hexane-1,3-5-tricarbonitrile *	13.935	hexane-1,3-5-tricarbonitrile *
14.282	pentane-1,3,5-tricarbonitrile	14.282	pentane-1,3,5-tricarbonitrile
15.389	hexane-1,3-5-tricarbonitrile	15.389	hexane-1,3-5-tricarbonitrile
**PANVDC-ATO/ZHS**		**PANVDC-ZHS**	
**Retention Time (min)**	**Compound Identified**	**Retention Time (min)**	**Compound Identified**
1.469	hydrogen chloride *	1.469	hydrogen chloride *
1.615	acetonitrile	1.615	acetonitrile
1.67	acrylonitrile *	1.67	acrylonitrile *
1.899	methylacrylonitrile *	1.899	methylacrylonitrile *
2.923	2,4-pentadienenitrile	2.923	2,4-pentadienenitrile
5.319~5.466	chloropyridine isomers *	5.319~5.466	chloropyridine isomers *
6.225	benzonitrile	6.225	benzonitrile
7.386	2-methylenepentanedinitrile *	7.386	2-methylenepentanedinitrile *
7.642	2-methylpentanedinitrile	7.642	2-methylpentanedinitrile
7.908	m-toluidine	7.908	m-toluidine
9.856	Antimony compound	-	-
10.551	isophthalonitrile	10.551	isophthalonitrile
11.877	1-methylnaphthalene	11.877	1-methylnaphthalene
13.898	hexane-1,3-5-tricarbonitrile	13.898	hexane-1,3-5-tricarbonitrile
14.282	pentane-1,3,5-tricarbonitrile	14.282	pentane-1,3,5-tricarbonitrile
15.389	hexane-1,3-5-tricarbonitrile	15.389	hexane-1,3-5-tricarbonitrile

* Major peaks.

**Table 4:** TGA and DTG results of the PANVDC fibers with flame retardants at a heating rate 20 °C/min to 800 °C under air conditions.

Sample	First Stage		Second Stage	
	TMR1 * (°C)	Mass Loss (%)	TMR2 * (°C)	Mass Loss (%)
PANVDC	255	35	611	64
PANVDC-ATO	224	41	576	56
PANVDC-ATO/ZHS	188	36	605	63
PANVDC-ZHS	187	29	640	65

* TMR1 and TMR2: Temperature at the maximum rate of mass loss (%/°C) in the first and second stages, respectively.

**Table 5:** Limiting oxygen index (LOI) values of the PANVDC films with the flame retardants.

Samples	LOI (%)
PANVDC	26.4
PANVDC-ATO	29.0
PANVDC-ATO/ZHS	31.0
PANVDC-ZHS	33.5

**Table 6:** Microscale combustion calorimetry (MCC) data of the PANVDC fibers with the flame retardants.

Samples	HRC (J/[gK])	PHRR (W/g)	T_PHRR_ (°C)	THR (kJ/g)
PANVDC	90	90.4	279	2.6
PANVDC-ATO	116	115.4	272	2.1
PANVDC-ATO/ZHS	12	10.8	418	1.5
PANVDC-ZHS	11	9.8	433	1.6

**Table 7:** The degree of crystal orientation in the PANVDC fibers with flame retardants.

Sample	PANVDC	PANVDC-ATO	PANVDC-ATO/ZHS	PANVDC-ZHS
Crystal orientation (%)	71.09	71.08	72.61	70.30

**Table 8:** Mechanical properties of the PANVDC fibers with the flame retardants.

Samples	Tenacity (g/den)	Linear Density (den)	Elongation (%)
PANVDC	4.42 (±0.25)	5.39 (±0.44)	12.52 (±0.34)
PANVDC-ATO	3.11 (±0.41)	5.31 (±0.45)	9.34 (±1.02)
PANVDC-ATO/ZHS	3.73 (±0.52)	5.39 (±0.42)	10.69 (±0.52)
PANVDC-ZHS	3.75 (±0.25)	5.81 (±0.19)	11.05 (±0.66)
